# Social cognition in first-degree relatives of bipolar disorder: Theory of Mind and nonverbal sensitivity

**DOI:** 10.1371/journal.pone.0246908

**Published:** 2021-03-02

**Authors:** Usue Espinós, Enrique G. Fernández-Abascal, Mercedes Ovejero, Guillermo Lahera

**Affiliations:** 1 Facultad de Psicología, Universidad Nacional de Educación a Distancia, Madrid, Spain; 2 Facultad de Psicología, Universidad Complutense de Madrid, Madrid, Spain; 3 Facultad de Medicina, Universidad de Alcalá de Henares, Madrid, Spain; National Institutes of Health, UNITED STATES

## Abstract

Social cognition might be impaired in first degree relatives (FDR) of BD but existing research shows controversial results about social cognitive impairments in this population. The aim of this study was to assess Theory of Mind (ToM) and nonverbal sensitivity in FDR of BD and compare the results with those of two groups of persons with remitted bipolar disorder (BD), type I and II, and a control group. Social cognitive ability was examined in first degree relatives of BD, with a biological parent, offspring or sibling diagnosed with the disorder. For this study, 37 FDRs of bipolar patients, 37 BD I, 40 BD II and 40 control participants were recruited. Social cognition was explored by means of the Reading the Mind in the Eyes Test and the MiniPONS. Results showed a significant impairment in FDR of BD in the ToM task, but not in nonverbal sensitivity. Performance of FDRs in social cognition is better than that of BDs (either type I or type II) but worse when compared with that of healthy individuals without a family history of psychiatric disorders. Nevertheless, no differences were found between BD I and BD II groups. Males and older participants showed a worse performance in all groups. Group family therapy with FDRs of BD might include training in the recognition of nonverbal cues, which might increase the understanding of their familiars with BD, in order to modify communication abilities.

## Introduction

Bipolar disorder (BD) is a psychiatric condition, characterized by extreme fluctuations in energy and mood [1]. Bipolar II (BD II) is distinguished from bipolar I (BD I) by the presence of hypomanic episodes, and is a milder condition than BD I, in relation with mood elevation [1]. With respect to the severity, for some authors, psychosocial impairment increases significantly in BD I with most increments in manic symptom severity [2]. Few studies have compared BD II with healthy controls, but findings suggest that BD II patients are at least so functionally disabled as BD I patients, and experience functional impairment in all domains, that continues after remission of symptoms [3]. Nevertheless, with regard to research on psychosocial functioning, BD II is not only understudied, but there is also scarce evidence about this question. Most research evaluating functional disability in BD is based on patients with BD I, with no comparison of the two groups, BD I and BD II [4, 5].

Social impairment can be observed in many BDs [6]. Evidence suggests that during euthymic periods, they also undergo interpersonal difficulties [7]. It is estimated that up to 60% of individuals do not fully recover after episodes [8] and only 38% of them achieve functional recovery after a manic phase [9]. BD can be profoundly disabling, and is associated with a substantial loss of work performance, and the consequent financial burden [10]. In relation with severity of BD, a lack of objectivity exists in the current diagnostic system to differentiate more severe patients. Some studies define severity of illness course as early onset of BD [11]; other authors consider illness severity of BD as having anxiety symptoms [12, 13], or correlation with familial psychiatric history: in most cases, with a first-degree relative with severe mental disorder [14, 15].

There is evidence indicating that social cognition deficits are present in patients with BD, even in the euthymic phase [16]. BD patients exhibit deficits in several social cognition domains, including emotional processing [17]. Social cognition refers to the psychological operations related to the perception and interpretation of social signals, that enable individuals to learn about the world, oneself, and the others [18]. A central process within this construct is Theory of Mind (ToM), defined as -the competence to interpret and predict other persons’ behavior by attributing mental states such as feelings, desires, beliefs, opinions and intentions, and the ability to share and recognize the emotions of others, to understand and predict their behavior [19]. There are divergent findings in social cognition studies of BD. Some studies found worse performance in BD I compared to BD II [20]. For other authors, both groups have a similar poor performance in social cognition tasks, compared to controls [21, 22]. Social cognition has been mostly investigated with BD through a ToM measure, the “Reading the Mind in the Eyes Test” (RMET) [23]. Many authors have found deficiencies in this population; remitted bipolar participants, when assessed with this tool, scored significantly lower, when compared to healthy controls [24–27].

Another aspect of social cognition is nonverbal sensitivity, that is, the ability to decode affective nonverbal cues in others [28]. One example of nonverbal sensitivity measure is the test MiniPONS [29], a test with ecological validity, which includes fundamental information such as movements of face, body and voice. This test presents scenes that are nearer to real life situations than static pictures. Expression of emotion through body language has not been extensively explored [30], as communication of emotions by means of dynamic body movements and gestures conveys specific information about emotion processing of others’ emotional states and intentions [31]. Variables such as age and gender differences have also been investigated in both tests, RMET and MiniPONS, with no conclusive results. Some studies have found significant female superiority in RMET [32, 33], other investigations have found that females do not score significantly higher than males [eg. 23, 34–37]. Nevertheless, there are numerous investigations that show that women obtain significantly higher scores in tests that assess the ability to identify emotional facial expressions [38–41]. Performance in RMET decreases with age [42]. Regarding gender in MiniPONS performance, women achieve significantly better results than men [29], they process nonverbal emotional information more efficiently than men and obtain better scores in all channels [43]. With respect to the relationship between age and nonverbal sensibility, this ability declines with aging [43].

Evidence from family, twin and adoption studies indicates a heritable component to BD [44], suggesting a substantial genetic contribution to disease etiology and an elevated risk of developing BD [45]. If these deficits were the phenotypic expression of genetic vulnerability to BD, healthy subjects with a genetic predisposition to BD would be expected to display the same deficits. Therefore, social cognitive dysfunction might be a possible endophenotype in BD. Research in social cognition of first-degree relatives of BD (FDR) is scant, there are findings that show that these individuals have a significant, but small impairment [46]. In this population, several different social cognitive tasks have been utilized across studies [47–49]. When measuring ToM in FDRs, with RMET, there are contradictory results. One study comparing offsprings with controls found significant deficits in this population [50]; another study did not find significant differences in unaffected adult FDRs of euthymic persons with BD [47]. Nonverbal sensitivity has not been measured in FDRs and only one study has been done with remitted BD, showing that these subjects performed significantly worse than the control group [51]. Given that the test MiniPONS has not been used before either with BDI or BD II population or with their first-degree relatives (comparing the three groups), this is an innovative aspect of this research. The aim of this study was to have a broader view of the difficulties of BDs and their families in the evaluation of ToM, assessing ToM with RMET and nonverbal sensitivity with MiniPONS, as useful measures of social cognition.

### The present study

The objective of this research was to study first- degree relatives (FDR) of BD in a particular domain, social cognition, and compare their performance to that of two groups of remitted BDs (including not only a group of BD I, but also another group of BD II), and a control group. To the best of our knowledge, no previous studies explored performance of FDRs, comparing them with patients with BD II in ToM tasks, nor in nonverbal sensitivity. Among social cognition measures, a ToM test was chosen, the RMET, a tool that tests the ability to recognize emotional expressions and complex cognitive mental states. Theory of Mind in bipolar disorder has mostly been measured with the test RMET. As the RMET is based on facial static pictures, the objective was to complement this task with another dynamic tool of social cognition that presents scenes that are closer to real life situations, the MiniPONS, to explore their ability in the perception of different dynamic nonverbal channels as face, body movements and voice. The aim was to have a broad spectrum of family members’ ability to recognize nonverbal cues, and to determine if BD I and BD II had worse performance than FDR. Specific hypotheses tested were: 1) social cognition deficits, measured with the two tests, are higher in patients with BD I or BD II than in FDR group; 2) when compared with healthy individuals without a family history of psychiatric disorders, results of FDRs are lower than those of the control group; 3) BD II participants do not have better results than BD I, and; 4) age and gender of participants affects the performance of all subjects; older participants and males perform worse in both tasks.

## Materials and methods

### Participants

The sample consisted of 154 persons: 37 FDRs of BD and, for comparison, 37 BD I, 40 BD II (both BD groups were in clinical remission) and 40 healthy controls. FDR and BD participants were recruited through self-help groups, and every FDR had only one first-degree biological sibling, offspring or parent who had a diagnosis of BD I or II. Collected data were age, gender, educational level, marital status, occupation, and diagnosis (BD I or BD II). The FDR sample comprised 37 individuals over the age of 25 (23 females and 14 males). Inclusion requirements criteria for the FDR group were: to have a first- degree familiar with a diagnosis of BD (I or II) and no current or past history of psychiatric or neurological illness, as well as no substance abuse. For BD groups, to enter the study, they should have been diagnosed with BD I or II and the requirement of having been euthymic at least during the previous three months.

BD is highly comorbid with addictions [52], Bipolar disorders are highly associated with alcohol use disorder [53], and generally, substance abuse is a major comorbidity in BD [54]. Lack of these two comorbidities was acknowledged in this study. Exclusion criteria for BD were as follows: patients with a (hypo)manic or depressive episode in the previous three months, alcohol abuse in the past six months or use of psychoactive substances during the same period. All BD I and BD II participants were receiving pharmacological treatment. Control group participants had no current or past psychiatric disorder. They were age and sex matched with FDR participants. Demographic characteristics are listed in Table 1.

**Table 1:** Demographic and clinical characteristics of the sample.

	BDI I (n = 37)		BD II (n = 40)		FDR (n = 37)		Control (n = 40)		F	df	p
**Age (Years)**	44.73±12.81		49.88±11.47		51.03±13.51		48.53±13.84		1.738	3, 149	.162
**Gender**	**n**	**%**	**n**	**%**	**n**	**%**	**n**	**%**	χ^2^	df	p
Female	22	59.46	22	55.00	23	62.16	24	60	.274	3	.966
Male	15	40.54	18	45.00	14	37.84	16	40			
**Medication BD**	**n**	**%**	**n**	**%**					χ^2^	df	p
Lithium	19	51.35	13	33.33					2.812	1	.094
Anticonvulsant	14	37.83	23	58.97					2.976	1	.084
Antipsychotic	22	59.45	12	30.76					6.765	1	**.009**
Antidepressant	2	5.4	8	20.51					3.622	1	.057

### Ethical statement

The study was approved by the Research Ethics Committee of Universidad Nacional de Educación a Distancia (Spain) and has been conducted according to the principles expressed in the Declaration of Helsinki. To participate, and after a thorough explanation of the study, all subjects provided written informed consent.

### Measures

Following informed consent, and in order to verify the inclusion and exclusion criteria, the MINI Neuropsychiatric interview (in its Spanish adaptation) [55], was administered to FDR and BD groups. To confirm euthymia, absence of depressive and manic symptoms in BD, was measured with the Beck Depression Inventory II (BDI II) [56], in its Spanish version [57], and the Young Mania Rating Scale (YMRS) [58], in its Spanish adaptation [59].

The cut-off score in the scales to assess euthymia was ≥ 30 on the BDI II and ≥ 7 on the YMRS. In the Spanish adaptation for BDI II, for non-clinical and clinical Spanish populations, the cut-off scores would be equal to or higher than 19 and 30 respectively, inasmuch as those scores would show specificities over 90% and positive predictive values of 61% [60].

### Social cognition assessment: ToM and nonverbal sensitivity

Two social cognition tasks were administered: the "Reading the Mind in the Eyes" (RMET) [23] to assess ToM, and the “MiniPONS” [29], to evaluate nonverbal sensitivity:

1. The Spanish version of the test RMET [61].
  This tool involves examining 36 facial pictures and measures the ability to recognize what other people are thinking or feeling. The images consist of 36 grayscale photographs of the eye region of faces, of pictures that show only the eyes area of males and females (equal number of male and female faces) that reflect complex mental states and social emotions (e.g., joking, surprised, contemplative). Below every photograph, there is a four-choice selection and the subjects have to choose one option (only one is the correct). 36 points is the highest score that can be achieved if all answers are correct. It has an approximate duration of 15 minutes, but there is no time limit to answer. A software application collects the stimuli and stores the responses. Reliability of this scale score for the present study was α = .71.
2. The test MiniPONS in its Spanish version [43]. MiniPONS is a dynamic test that measures individual differences in the ability to recognize emotions, interpersonal attitudes and intentions, expressed through different nonverbal channels. MiniPONS consists in a set of short 64 video clips in black and white (plus three examples), that feature a woman with manipulated negative and positive emotional tone of facial expressions, body language, and voice. MiniPONS is composed by different expressive channels, in which all stimuli are grouped into a 2 x 2 design that combines affective valence and dominance: half of the stimuli show positive affect and the other half, negative. Similarly, half of the stimuli express dominant attitudes and the rest, submissive. It is administered through a computer application that presents the stimuli and records the responses (the total score). The response procedure is as follows: the video clip is present for two seconds, it disappears, and two possible answer options are shown in the screen. The participant must choose one of them, the one he thinks correct as to what the woman in the video is expressing. Once the subject has chosen the answer, the next video comes up. Total scores for each dimension were computed and reliability was near α = .70.

### Data analysis

The analysis performed to verify the endpoints of this study was a multiple linear regression analysis, to examine if social cognition differed among groups. Independent variables were group, gender and age, and dependent variables were the performance in the MiniPONS and RMET. To check if there was collinearity between predictors, several collinearity tests were computed. After checking that collinearity was non-significant, the independent variables were introduced in the model one-by-one, starting with the group, following by gender, and finally, age was the last variable included in the model. Significant level for all the regression analyses was .05. The analysis was carried out by means of R software [62].

## Results

**Fig 1:**
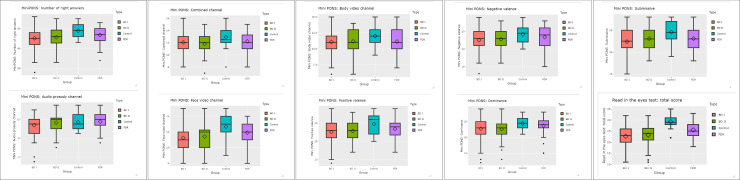
Differences between groups.

**Fig 2:**
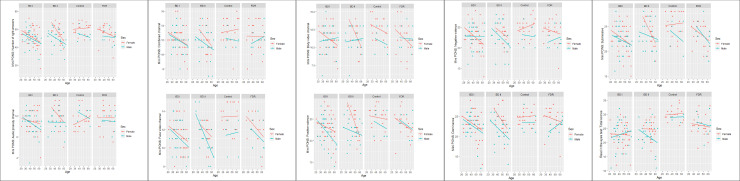
Scatterplots: Role of group, sex and age in the study variables.

Descriptive statistics and regression analysis are shown in Tables 2 and 3 and Figs 1 and 2.

**Table 2:** Descriptive statistics.

Variable	Group	N	Mean	Median	SD	Min	Max	Skew	Kurt
MiniPONS Number of right answers	Control	40	48.93	49.00	3.68	43.00	55.00	-0.03	-1.30
	BD I	37	44.70	45.00	4.75	33.00	54.00	-0.23	-0.32
	BD II	40	44.88	44.50	4.79	33.00	55.00	-0.08	-0.28
	FDR	37	46.78	47.00	4.31	34.00	53.00	-0.78	0.42
Audio prosody channel	Control	40	11.37	11.00	1.71	9.00	15.00	0.44	-0.74
	BD I	37	10.70	11.00	2.15	3.00	14.00	-1.17	2.42
	BD II	40	11.00	11.50	1.89	6.00	15.00	-0.42	0.00
	FDR	37	11.51	12.00	1.95	7.00	15.00	-0.54	-0.51
Combined channel	Control	40	12.90	12.50	1.75	8.00	16.00	-0.38	0.03
	BD I	37	11.95	12.00	1.49	9.00	16.00	0.38	-0.14
	BD II	40	11.57	12.00	1.93	7.00	15.00	-0.38	-0.23
	FDR	37	12.22	12.00	1.86	8.00	15.00	-0.38	-0.36
Face video channel	Control	40	12.67	13.00	1.86	9.00	15.00	-0.42	-0.71
	BD I	37	11.32	12.00	1.80	8.00	15.00	0.05	-1.02
	BD II	40	11.28	11.00	1.72	8.00	15.00	-0.04	-0.45
	FDR	37	11.92	12.00	1.61	8.00	14.00	-0.54	-0.64
Body video channel	Control	40	12.00	12.00	1.91	9.00	15.00	-0.17	-1.12
	BD I	37	10.73	11.00	1.81	6.00	13.00	-0.71	-0.26
	BD II	40	11.03	11.00	1.85	6.00	14.00	-0.39	-0.40
	FDR	37	11.14	11.00	1.89	7.00	15.00	0.03	-0.49
Positive Valence	Control	40	24.77	26.00	2.75	20.00	29.00	-0.38	-1.22
	BD I	37	22.38	23.00	3.08	14.00	28.00	-0.50	-0.03
	BD II	40	22.55	22.50	2.79	17.00	28.00	0.25	-0.76
	FDR	37	23.43	24.00	2.62	17.00	29.00	-0.22	-0.37
Negative Valence	Control	40	24.17	24.50	2.42	20.00	29.00	-0.03	-1.05
	BD I	37	22.32	23.00	2.89	16.00	28.00	-0.22	-0.90
	BD II	40	22.32	23.00	3.13	16.00	28.00	-0.18	-0.53
	FDR	37	23.35	24.00	2.74	15.00	28.00	-0.63	0.25
Dominant	Control	40	24.37	24.50	2.06	21.00	28.00	0.16	-1.02
	BD I	37	22.16	22.00	3.05	12.00	27.00	-0.86	1.40
	BD II	40	22.32	22.50	3.36	13.00	29.00	-0.29	0.30
	FDR	37	23.70	24.00	2.63	15.00	28.00	-1.09	1.58
Submissive	Control	40	24.57	24.50	2.76	19.00	28.00	-0.38	-0.92
	BD I	37	22.54	23.00	2.74	16.00	28.00	-0.39	-0.38
	BD II	40	22.55	22.00	2.67	18.00	28.00	0.50	-0.68
	FDR	37	23.08	23.00	2.64	18.00	28.00	-0.33	-0.99
RMET: Total score	Control	40	29.23	29.00	2.80	22.00	35.00	-0.65	1.11
	BD I	37	22.41	22.00	3.94	12.00	30.00	-0.51	0.05
	BD II	40	23.05	24.00	4.55	11.00	32.00	-0.75	0.57
	FDR	37	25.54	25.00	3.80	18.00	33.00	0.18	-0.53

Note. FDRs perform significantly worse than the control group in RMET.

**Table 3:** Multiple linear regression in the prediction of the performance in MiniPONS and RMET tests.

Variable	Predictor	Estimate	95% CI	*t*	*P*	$r_{adj}^{2}$	*F*	*P*
MiniPONS Number of right answers	BD I	-4.570	(-6.421, -2.718)	-4.877	**< .001**	.109	7.228	**< .001**
	BD II	-3.531	(-5.333, -1.730)	-3.873	**< .001**			
	FDR	-1.865	(-3.700, -.030)	-2.008	.046			
	Gender	-2.068	(-3.398, -.739)	-3.074	**< .001**	.168	8.747	**< .001**
	Age	-.108	(-.160; -.055)	-4.060	**< .001**	.247	11.02	**< .001**
	Intercept	54.94	(52.047, 57.833)	37.53	**< .001**	-	-	-
Audio prosody channel	BD I	-.836	(-1.708, .035)	-1.897	.060	.014	1.706	.168
	BD II	-.505	(-1.352, .343)	-1.177	.241			
	FDR	-.013	(-.876, .850)	-.030	.977			
	Gender	-.263	(-.888, .362)	-.831	.407	.012	1.467	.215
	Age	-.003	(-.028, .022)	-.253	.801	.001	1.179	.322
	Intercept	11.787	(10.427, 13.148)	17.116	**< .001**	-	-	-
Combined channel	BD I	-.820	(-1.599, -.040)	-2.078	**.004**	.037	2.935	**.035**
	BD II	-1.061	(-1.819, -.303)	-2.675	**.006**			
	FDR	-.479	(-1.251, .294)	-1.225	.224			
	Gender	-.651	(-1.210, -.091)	-2.297	**.023**	.068	3.795	**.006**
	Age	-.013	(-.036, .008)	-1.249	.214	.072	3.359	**.007**
	Intercept	13.654	(12.437, 14.872)	22.158	**< .001**	-	-	-
Face video channel	BD I	-1.513	(-2.214, -.813)	-4.267	**< .001**	.072	4.957	**.003**
	BD II	-1.148	(-1.830, -.466)	-3.328	**.001**			
	FDR	-.597	(-1.292, .097)	-1.699	.092			
	Gender	-.637	(-1.140, -.134)	-2.503	**.013**	.117	6.045	**< .001**
	Age	-.054	(-.074, -.034)	-5.350	**< .001**	.255	11.46	**< .001**
	Intercept	15.504	(14.410, 16.599)	27.985	**< .001**	-	-	-
Body video channel	BD I	-1.400	(-2.211, -.589)	-3.412	**< .001**	.040	3.097	**.029**
	BD II	-.817	(-1.606, -.028)	-2.046	**.043**			
	FDR	-.776	(-1.580, .028)	-1.908	.058			
	Gender	-.518	(-1.099, .065)	-1.757	.081	.061	3.480	**.009**
	Age	-.037	(-.060, -.014)	-3.175	**.002**	.115	4.969	**< .001**
	Intercept	13.994	(12.727, 15.260)	21.827	**< .001**	-	-	-
Positive valence	BD I	-2.386	(-3.566, -1.205)	-3.992	**< .001**	.058	4.153	**.007**
	BD II	-1.603	(-2.752, -.454)	-2.757	**.007**			
	FDR	-.854	(-2.045, .316)	-1.443	.151			
	Gender	-.900	(-1.748, -.053)	-2.099	**.038**	.091	4.820	**.001**
	Age	-.080	(-.113, -.046)	-4.697	**< .001**	.203	8.814	**< .001**
	Intercept	28.691	(26.846, 30.536)	30.735	**< .001**	-	-	-
Negative valence	BD I	-2.184	(-3.417, -.951)	-3.501	**< .001**	.071	4.887	**.002**
	BD II	-1.929	(-3.128, -.729)	-3.177	**.002**			
	FDR	-1.011	(-2.232, .211)	-1.635	.104			
	Gender	-1.168	(-2.053, -.283)	-2.608	**.010**	.112	5.811	**< .001**
	Age	-.028	(-.063, .007)	-1.599	.112	.121	5.209	**< .001**
	Intercept	26.249	(24.333, 28.175)	26.932	**< .001**	-	-	-
Dominant	BD I	-2.187	(-3.467, -.906)	-3.375	**< .001**	.059	4.192	**.007**
	BD II	-1.596	(-2.841, -.350)	-2.531	**.012**			
	FDR	-.355	(-1.624, .914)	-.553	.581			
	Gender	-1.286	(-2.205, -.367)	-2.764	**.006**	.109	5.668	**< .001**
	Age	-.052	(-.088, -.015)	-2.815	**.006**	.148	6.330	**< .001**
	Intercept	27.185	(25.185, 29.186)	26.857	**< .001**	-	-	-
Submissive	BD I	-2.350	(-3.497, -1.203)	-4.049	**< .001**	.082	5.505	**.001**
	BD II	-1.898	(-3.017, -.781)	-3.356	**.001**			
	FDR	-1.475	(-2.613, -.337)	-2.562	**.011**			
	Gender	-.804	(-1.626, .019)	-1.930	.055	.107	5.534	**< .001**
	Age	-.057	(-.089, -.024)	-3.454	**< .001**	.168	7.140	**< .001**
	Intercept	27.750	(25.968, 29.533)	30.768	**< .001**	-	-	-
RMET: Total score	BD I	-7.368	(-9.022, -5.713)	-8.800	**< .001**	.348	28.270	**< .001**
	BD II	-6.217	(-7.827, -4.607)	-7.633	**< .001**			
	FDR	-3.881	(-5.521, -2.242)	-4.678	**< .001**			
	Gender	-1.400	(-2.588, -.213)	-2.331	**.021**	.373	23.750	**< .001**
	Age	-.062	(-.109, -.015)	-2.603	**.010**	.396	21.090	**< .001**
	Intercept	33.106	(30.522, 35.690)	25.314	**< .001**	-	-	-

*Note*. CI: Confidence interval. Reference category for group = ‘control’. Reference category for group = ‘female’.

Regression analysis demonstrated that, in the RMET, FDRs results were significantly worse than those of the control group for the number of right answers variable, in the total score of this test, and BD II group had not better results than BD I. Age was negatively related to performance, and males made more mistakes than females in all groups. The percentage of associated variance was equal to 39.60%. In the MiniPONS, regression analysis showed that, in FDRs performance, there were not significant differences with control group. Patients with BD (I and II) had worse performance than controls in the number of correct responses achieved in this test. The percentage of associated variance was equal to 24.70%.

With respect to the MiniPONS channels, in the combined channel, there were no differences between FDRs and control group, and men scored lower than women (percentage of associated variance was equal to 7.20%). The same results, no differences between controls and FDRs, and men´s lower scores, were found in the face-video (percentage of associated variance 25.50%), body-video (percentage of variance 11.50%), dominant (percentage of associated variance 14.80), submissive (percentage of associated variance 16.80%), positive (percentage of associated variance 20.30%) and negative valence (percentage of associated variance 12.10%) channels. BD groups (I and II) had worse results than controls and FDRs in all channels, except in the audio prosody channel (no differences between groups, age and gender).

## Discussion

This study investigated social cognition abilities in FDRs of BD as a risk for developing BD in this population. Two tools were used: RMET and MiniPONS. Consistent with the literature, the results supported the hypothesis that both BDs and their first-degree relatives had a deficit in social cognition. With regard to differences in social cognition between BD I and BD II, little research exists with respect to differences in social cognition between these two groups, inasmuch as most studies are carried out with BD I or mixed samples of BD I and BD II patients. Nevertheless, when they are explored with a ToM task, both groups seem to have similar impairments [22]. The second hypothesis of this study, predicting that BD II would not have better scores than BD I in both tests, was also confirmed: no significant differences in the results between BD I and BD II were found, both groups have the same impairment.

With respect to the results in the positive valence channels of the MiniPONS, in the present study, BD groups had lower performance than control participants. Research exists that shows that BD is associated with persistently heightened positive emotional responses across contexts, compared with healthy controls [63, 64]. Gruber [65], studying BD I patients, found evidence that positive affect is more activated in this clinical group, comparing them with healthy population. In this study BD groups (I and II) and FDRs did not show this impairment, their performance in this variable was similar as that of the control group.

The results of this research showed that FRDs of BD, in the performance of the RMET, had a significant impairment in the recognition of complex mental states. FDRs performance was worse than that of control group and better than BDs (either type I or type II). However, with the test MiniPONS, no significant differences were found between FDRs and controls scores in this test. The results of this study showed that, RMET discriminated better between BDs (BD I and BD II groups) and their first-degree relatives, as BD familiars presented significative differences with controls in RMET, but not in MiniPONS. According to the expectations, the hypothesis predicting an intermediate performance of BD relatives between controls and BDs, was confirmed; they performed better than controls, but worse than BDs. These results could be understood as an empathy deficit of FDRs, that might have consequences for overall social functioning. This may negatively influence in the understanding of familiars diagnosed with BD and thus, have a repercussion in family relationships. This empathy deficit may be related to expressed emotion in FDR, that is, a measure of criticism, hostility and/or emotional overinvolvement in caregiving relatives when describing interactions with the patient [66, 67]. Deficits in social cognition may lead to impaired communication, and research has demonstrated that FDRs high expressed emotion communication is associated with risk of mood relapse among adults with BD [68–70]. This lack of understanding may also contribute to difficulties in maintaining adequate familial relationships, inasmuch as empathic skills are essential for successful social interactions [71]. Little research exists regarding differences in social cognition between BD II and BD II, inasmuch as most studies are carried out with BD I or mixed samples of BD I and BD II patients. Both groups seem to have similar impairments when they are explored with a ToM task [22]. We predicted that BD II would not have better scores than BD I in both tests, and this hypothesis was also confirmed. The prediction that male gender and age of participants would affect the performance of participants in both tests, was also confirmed in this study. Results showed that men and older participants (from all groups) obtained lower results in RMET and in most channels of MiniPONS.

The model of emotional competence [72]. emphasizes the importance of the ability to perceive emotional signals that facilitate the adaptation of the individual to constantly changing environments. Social cognitive abilities are crucial for effective interpersonal functioning. In this research, results show that FDRs were partially competent in the decoding of nonverbal signals, they were not very proficient in the understanding of other´s internal states. Group psychoeducation in order to modify unproductive cycles of family interaction is a suggestion of some authors as Vieta [73]. One of the most relevant family interventions is that of Miklowitz et al. [67], who suggest the psychoeducational model as a type of psychosocial family intervention that may reduce the level of symptom severity or functional impairment. Family psychoeducation may increase families’ abilities to diminish the number of relapses as well as to enhance BD patients’ adherence to pharmacotherapy [74, 75]. In BD, possibly family therapy may be beneficial as adjuncts to pharmacological maintenance treatments [67]. Family Focused Treatment aims to reduce the high levels of stress and conflict in the families of bipolar patients, thereby improving the patient’s illness course [76]. It has increased positive and decreased negative family communication, and BD patients who had parents who were negative, critical, or guilt-inducing in their interactions with the patient, had a 94% chance of having an illness recurrence in the 9 months after a hospitalization [67].

In the last years, in BD, the focus has also moved from clinical remission to functional recovery [77]. The concept of recovery, not only symptomatic (low scores on ratings of mania and depression that indicate near-absence of symptoms), but also functional recovery, is a goal to achieve in these patients. Thereby, psychosocial interventions with BD can directly help to prevent relapse, and social cognitive treatment to those with an actual diagnosis of bipolar disorder, may be desirable. Psychological treatments are cognitive-behavioral therapy, psychoeducation, interpersonal and social rhythm therapy, and family intervention [78]. A suggestion is, that treating BD disorder within a familial context, enhancing FDRs competences in social cognition abilities, in the inference of BDs internal states might increase the ability to identify communication conflicts due to a poor understanding of nonverbal signals. Adjunctive family interventions are beneficial on BD outcomes and caregivers well-being. Those interventions lead to decrease the patients’ risk of recurrences and functional psychosocial impairment [79]. Group family therapy with FDRs of BD may include training in the recognition of nonverbal cues, which might help them to achieve a better understanding of their familiars with BD, in order to modify communication abilities. This competence, learning not to attribute nonverbal patient´s behaviors as negative, may improve family relationships, a better management of their relatives with BD and acquisition of more positive patters of interaction.

Among the main limitations of this research, there is the fact that FDRs were not matched with the BDs participants. A future prospective may be to conduct a study with a FDR group, in which every individual could be matched with their familiar with BD. Another limitation is the women´s ratio in FDR group, that was higher than that of men, which leads to a possibility of a gender bias. There is also the fact that in the choice of social cognition tasks, in this research, RMET and MiniPONS were chosen, and there is a large number of available tasks that evaluate social cognition domains. Another limitation is the relatively small size of the sample, which could have reduced the significance of some results. Moreover, cognitive functioning of BDs has not been scored in this study. As a future prospect, in social cognition research, a suggestion is to measure the cognitive profile of BDs. In this research, all BD participants were medicated. If medication of BDs might have an influence on the results of this study, was not investigated, and establishing an impact of pharmacology on cognition in BD sample, is a complex issue. Confirmation of this will require further investigation.

## Conclusions

This research studied ToM and nonverbal sensitivity in FDR of BD. Two tasks were chosen, the RMET, a tool that tests the ability to recognize emotional expressions and complex cognitive mental states, and MiniPONS, a dynamic test that measures facial expressions, body language, and voice intonation. Results showed significative impairment in FDR in the ToM task, but not in nonverbal sensitivity, they did not perform better than BDs. A suggestion is that, treating BD disorder within a familial context, enhancing FDRs competences in social cognition abilities, may improve family relationships and a better management of their relatives with BD.

## Supporting information

S1 Dataset (CSV)
